# MicroRNA-27b inhibition promotes Nrf2/ARE pathway activation and alleviates intracerebral hemorrhage-induced brain injury

**DOI:** 10.18632/oncotarget.19974

**Published:** 2017-08-07

**Authors:** Wenzhe Xu, Feng Li, Zhiguo Liu, Zhenkuan Xu, Bin Sun, Jingwei Cao, Yuguang Liu

**Affiliations:** ^1^ Department of Neurosurgery, Qilu Hospital and Brain Science Research Institute of Shandong University, Jinan 250012, P.R. China; ^2^ Department of Neurosurgery, People’s Hospital of Zhangqiu, Jinan 250200, P.R. China

**Keywords:** intracerebral hemorrhage, microRNA-27b, Nrf2, oxidative stress, neuroinflammation

## Abstract

Oxidative stress and neuroinflammation are the key factors leading to secondary brain injury after intracerebral hemorrhage (ICH). We investigated the effects of miR-27b, an oxidative stress-responsive microRNA, on ICH-induced brain injury in rats. The ICH model was induced by intracerebral injection of collagenase. Following ICH, miR-27b expression in the striatum was reduced, whereas expression of Nrf2 mRNA and protein was increased. In PC12 cells, overexpression of miR-27b reduced expression of Nrf2, Hmox1, Sod1 and Nqo1, while miR-27b inhibition had the opposite effects. Dual luciferase reporter assays showed that Nrf2 mRNA was a direct target of miR-27b. Intracerebroventricular injection of miR-27b antagomir and transfection of miR-27b inhibitor inhibited endogenous miR-27b in rats and PC12 cells, respectively. MiR-27b antagomir promoted activation of the ICH-induced Nrf2/ARE pathway and reduced the lipid peroxidation, neuroinflammation, cell death and neurological deficits otherwise seen after ICH. In PC12 cells, the miR-27b inhibitor diminished iron-induced oxidative stress, inflammation and apoptosis, and those effects were blocked by Nrf2 knockdown. These results demonstrate that miR-27b inhibition alleviates ICH-induced brain injury, which may be explained in part by its regulation on the Nrf2/ARE pathway.

## INTRODUCTION

Intracerebral hemorrhage (ICH) is a devastating subtype of stroke with high morbidity and mortality, accounting for about 15% of deaths from stroke [1]. Even after surviving the initial ictus, ICH patients will experience varying degrees of neurological dysfunction and require long-term hospitalization and rehabilitation [2]. The pathophysiologic process after ICH can be divided into two phases: the primary injury caused by the mass effect of intraparenchymal hematoma and the secondary brain damage resulting from oxidative stress (OS) and neuroinflammation in the perihematomal area [3, 4]. The secondary damage may be disproportionally important in ICH [5]. In the secondary brain injury, divalent iron ions, generated from hemoglobin breakdown, can cause OS by promoting free radical formation [6]. OS could subsequently induce expression of proinflammatory cytokines and also activate nuclear factor-κB (NF-κB), a key regulator of inflammatory responses [7, 8]. Additionally, proinflammatory cytokines may also induce production of free radicals, thereby creating a vicious positive feedback cycle [9]. Moreover, our previous studies demonstrated that NF-κB activation correlated with cell death in the perihematomal area and with the outcome of ICH patients [10, 11].

MicroRNAs (miRs) are a family of small noncoding RNA molecules, ranging in size from 19 to 25 nucleotides, which bind to the 3’-untranslated region (3’-UTR) of their target mRNAs, targeting them for degradation or inhibition of their translation, thereby suppressing protein expression at the posttranscriptional level [12, 13]. MiRs are involved in a wide range of biological processes, including cell differentiation and development, metabolism, proliferation and apoptosis [14, 15]. MiR-27b, in particular, was shown to be OS-responsive [16, 17]. It is downregulated in the livers of mice exposed to total body irradiation [18], and in auditory cells treated with different concentrations of tert-butyl hydroperoxide (t-BHP) [19]. In mouse macrophages, exposure to hydrogen peroxide induced OS and downregulated the expression of miR-27b [20]. In addition, miR-27b is an abundant neuronal miR implicated in numerous OS-related neurological disorders [21–23]. However, little attention has been paid to the regulatory effects of miR-27b on the anti-oxidative signaling pathways involved in ICH.

The nuclear factor erythroid 2-related factor 2 (Nrf2) is a master regulator of the anti-oxidative response and a potential target of miR-27b, with high scores and low mean free energies in three miRNA target prediction tools (TargetScan, miRanda and miRDB). Under normal conditions, Nrf2 is anchored by Kelch-like ECH-associated protein-1 (Keap1), which constitutively targets Nrf2 for ubiquitination and proteasomal degradation. When stimulated by oxidative and xenobiotic agents, Keap1 is inactivated and the ubiquitination stops, which leads to the accumulation of newly synthesized Nrf2 and its activation. Then the Nrf2 rapidly translocates to the nucleus and binds to the antioxidant response element (ARE), a cis-regulatory element sequence (5’-GTGACnnnGC-3’) in the promoter regulatory regions of the Nrf2-regulated genes, and promote expression of a broad range of antioxidants and detoxifying enzymes, including heme oxygenase 1 (Hmox1), superoxide dismutase 1 (Sod1), glutathione transferases (GSTs), glutathione peroxidase 1 (Gpx1), and NAD(P)H quinone oxidoreductase-1 (Nqo1), among others. [24–27]. In addition to its anti-oxidative effect, Nrf2 exerts anti-inflammatory effects through inhibition of NF-κB [28], and a growing body of evidence suggests that upregulation of Nrf2-regulated genes suppresses inflammatory responses [29–31]. Through its effects as an anti-oxidative and anti-inflammatory protein, Nrf2 appears to protect the brain from damage caused by ICH [32, 33]. Given that Nrf2 is a likely target of miR-27b, in the present study we examined the actions of miR-27b in a rat model of ICH-induced brain injury and in an *in vitro* iron toxicity model using PC12 cells.

## RESULTS

### Time patterns and negative correlation of miR-27b and Nrf2 expressions in ICH rats

**Figure 1:**
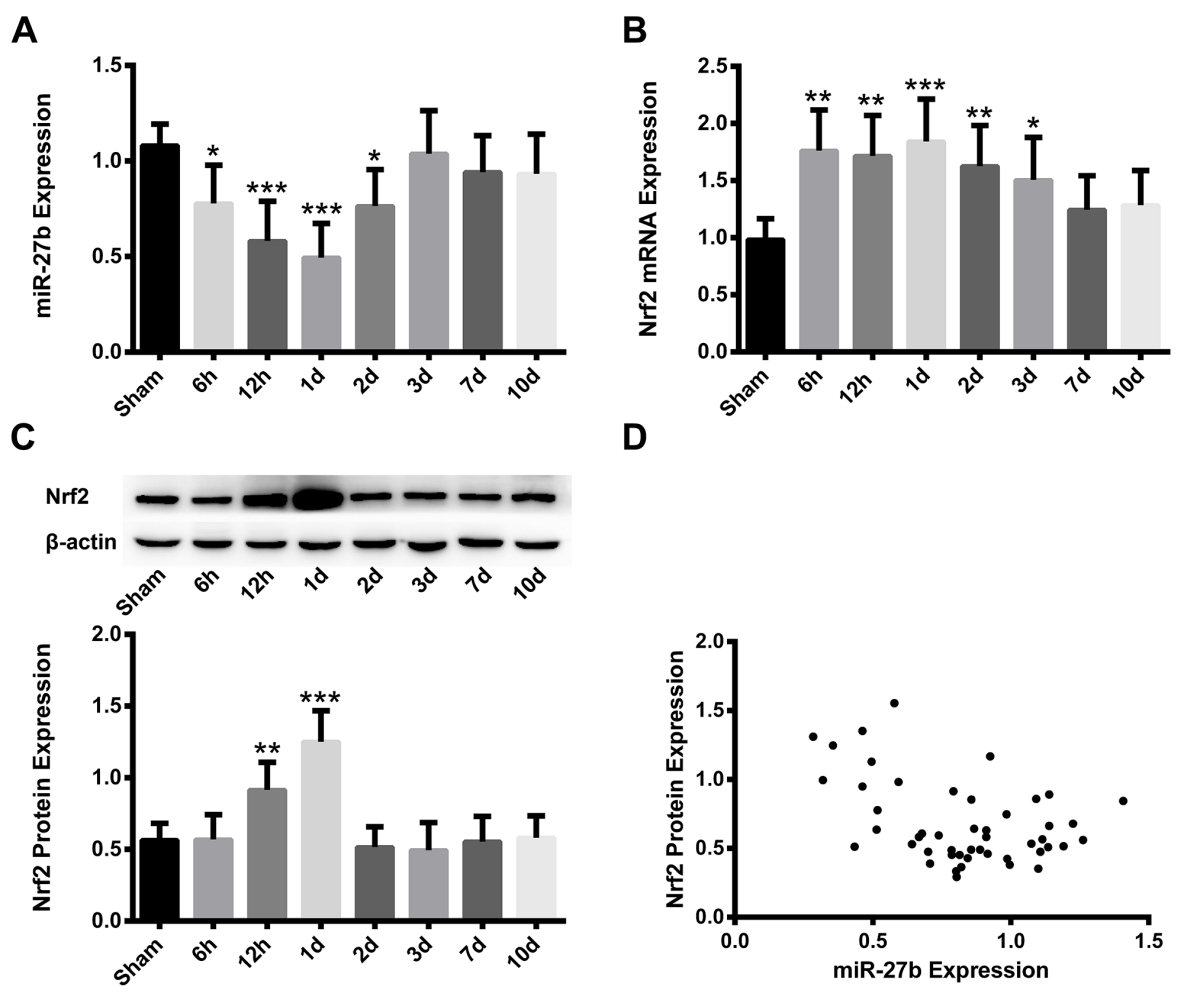
Time patterns and negative correlation of miR-27b and Nrf2 expression in ICH rats Analysis of qRT-PCR data for the expressions of **(A)** miR-27b and **(B)** Nrf2 mRNA. **(C)** Immunoblots and immunoblotting analysis of Nrf2 protein. **(D)** Scatter plot of miR-27b and Nrf2 protein expression (r=-0.4314, p<0.001). Data are presented as the mean ± SD (n=6). *p<0.05, **p<0.01, ***p<0.001 vs sham group.

Quantitative real-time polymerase chain reaction (qRT-PCR) and Western blot analysis were used to determine the time course of miR-27b, Nrf2 mRNA and protein expression at 6 h, 12 h, 1 d, 2 d, 3 d, 7 d, 10 d after ICH. The expression of miR-27b decreased as early as 6 h (p<0.05) after ICH and gradually reached its minimum at 1 d (p<0.001), followed by a progressive increase at 2 d (p<0.05) and was restored to normal levels at 3-10 d (p> 0.05) (Figure 1A). Meanwhile, Nrf2 mRNA expression increased significantly at 6 h-12 h (p<0.01), and peaked at 1 d (p<0.001), followed by gradual decrease at 2 d (p<0.01) and 3 d (p<0.05), then returned to control level at 7-10 d (p> 0.05) (Figure 1B). The Nrf2 protein levels were detected using Western blot analysis. The expression of the Nrf2 protein was elevated at 12 h (p<0.01), reaching peak levels at 1 d (p<0.001), followed by a sharp decrease to normal levels at 2-10 d (p> 0.05) (Figure 1C). The expression of miR-27b and the Nrf2 protein after ICH were negatively correlated (r= -0.4314, p<0.01) (Figure 1D). In addition, parallel time patterns of miR-27b and Nrf2 expressions were observed in the iron toxicity model in PC12 cells (Supplementary Figure 1).

### MiR-27b modulation regulated the expressions of Nrf2 and its downstream enzymes in PC12 cells

**Figure 2:**
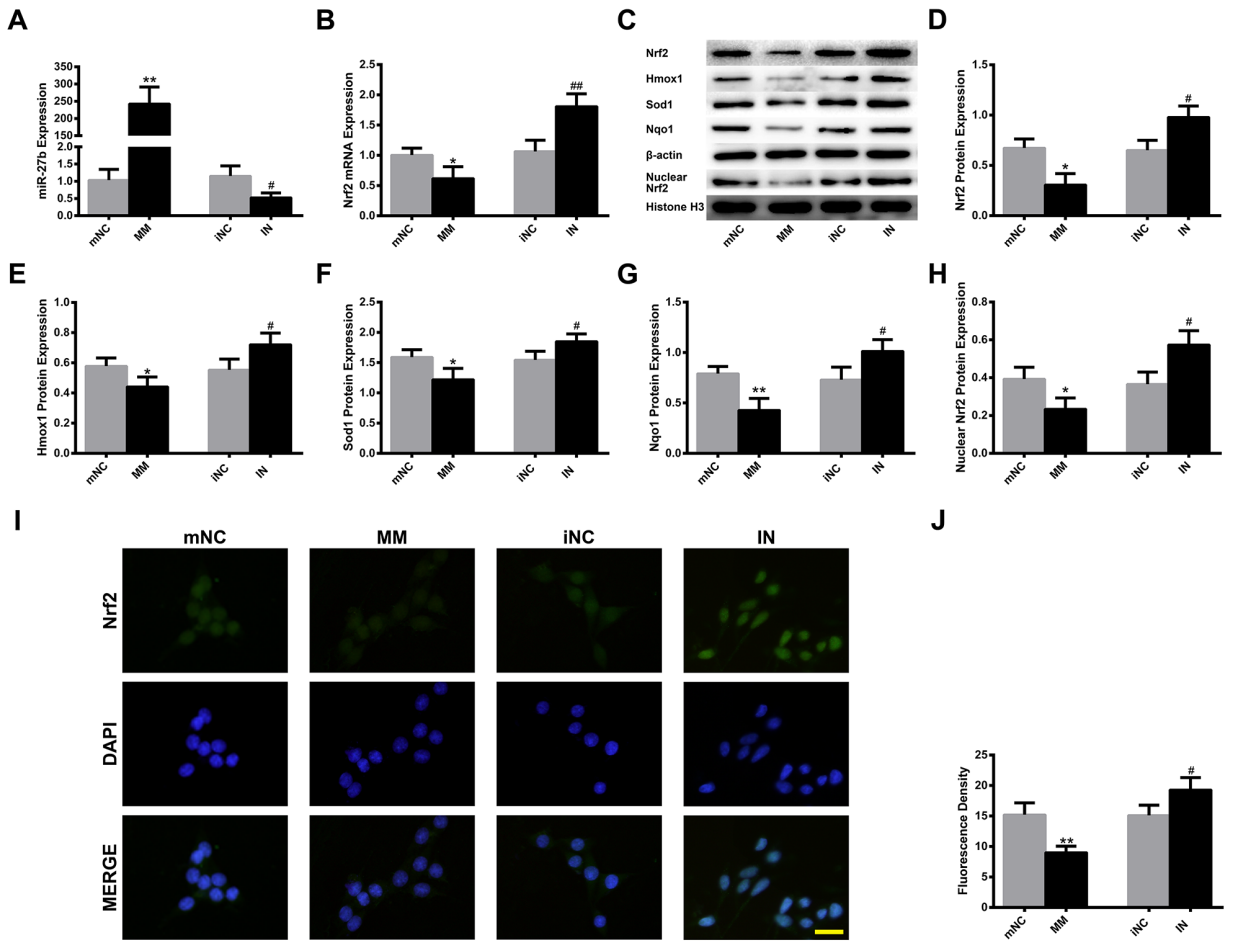
MiR-27b modulation regulates the expressions of Nrf2 and its downstream enzymes in PC12 cells PC12 cells were transfected with miR-27b MM, mNC or IN, iNC and incubated for 36 h. Cells were then harvested for qRT-PCR and Western blot, or fixed for immunofluorescence. Analysis of qRT-PCR data for the expressions of **(A)** miR-27b and **(B)** Nrf2 mRNA. **(C)** Immunoblots of Nrf2, Hmox1, Sod1, Nqo1 and nuclear Nrf2. Immunoblotting analysis of **(D)** Nrf2, **(E)** Hmox1, **(F)** Sod1, **(G)** Nqo1 and **(H)** nuclear Nrf2. **(I)** Immunofluorescence staining with anti-Nrf2 (green) in PC12 cells. Nuclei were counterstained with DAPI (blue). Scale bar=20 μm. **(J)** Quantification of fluorescence density. Data are presented as the mean ± SD (n=3). *p<0.05, **p<0.01 vs mNC, #p<0.05, ##p<0.01 vs iNC.

MiR-27b mimics (MM), MM negative control (mNC), IN, IN negative control (iNC) were transfected into PC12 cells and incubated for 36 h. MiR-27b MM dramatically enhanced the miR-27b level to about 235 times compared to the mNC group (p<0.01). Meanwhile, the miR-27b IN effectively decreased the miR-27b level (p<0.05) (Figure 2A). In addition, miR-27b overexpression significantly suppressed the expression of the Nrf2 mRNA and the protein levels of Nrf2, Hmox1, Sod1, Nqo1 and nuclear Nrf2 (all p<0.05), whereas miR-27b IN had the opposite effects (all p<0.05) (Figure 2B–2H). The Nrf2 expression was simultaneously examined by immunofluorescence, which showed that Nrf2 distributed throughout the cells, as shown in Figure 2I. Moreover, the fluorescence density was decreased after MM treatment (p<0.01), and increased after IN transfection (p<0.05) (Figure 2J).

### MiR-27b directly targeted the 3’-UTR of Nrf2 mRNA

**Figure 3:**
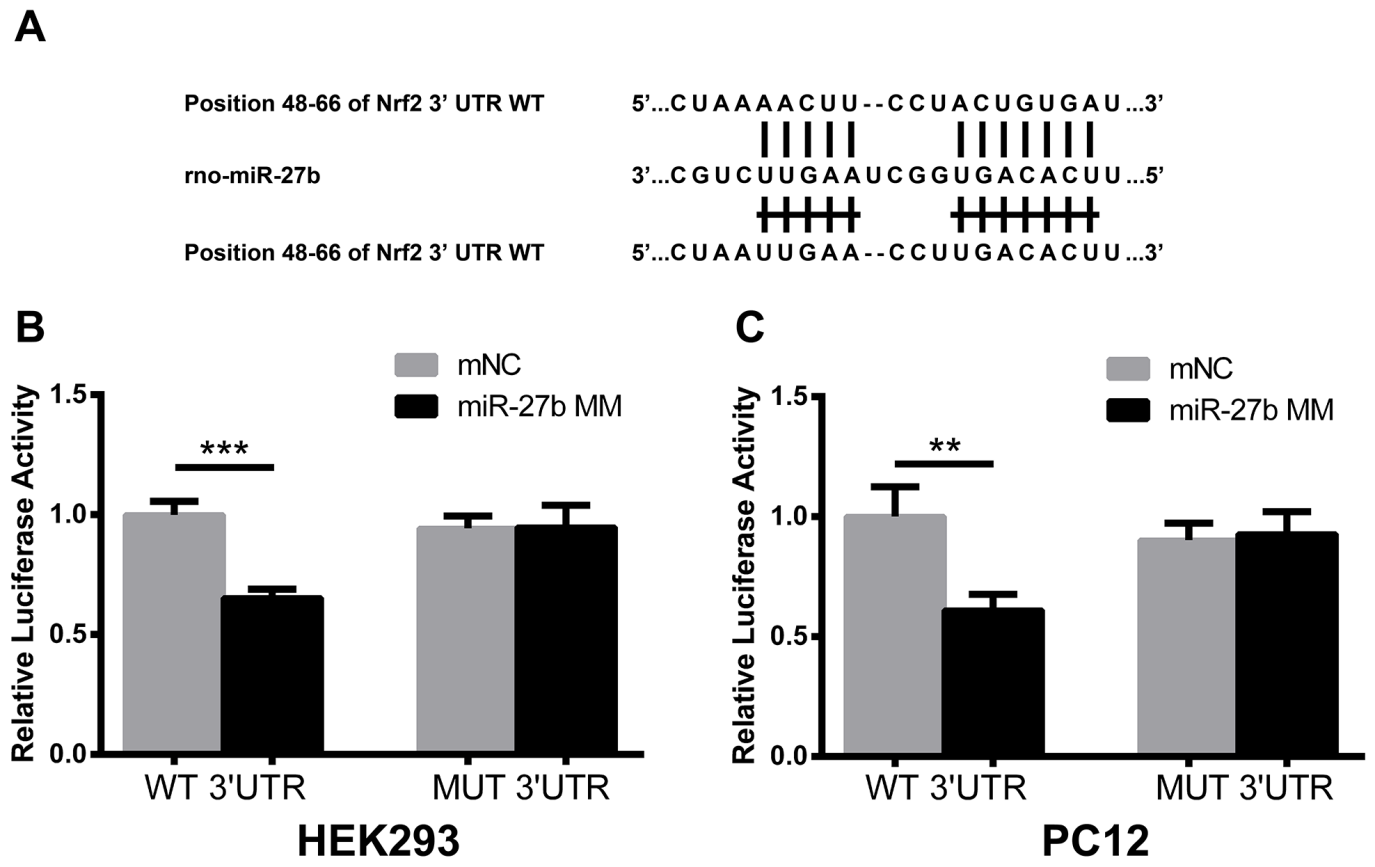
MiR-27b directly targeted the 3’-UTR of Nrf2 mRNA **(A)** A rat Nrf2 3’-UTR fragment containing wild-type (WT) or mutant-type (MUT) of miR-27b binding sites was cloned downstream of the luciferase reporter gene. Mutations were generated in the Nrf2 3’-UTR sequences complementary to the seed region of miR-27b, as indicated. Luciferase activity was analyzed at 48 h after HEK293 **(B)** and PC12 **(C)** cells were transfected with WT or MUT reporters, co-transfected with miR-27b MM or mNC. Activities are shown as the percentage relative to the cells transfected with mNC, which was set as 1. Data are presented as the mean ± SD (n=3). **p<0.01, ***p<0.001.

A dual-luciferase reporter assay was performed to determine whether Nrf2 mRNA was a direct target of miR-27b. The fragments of Nrf2 mRNA 3’-UTR containing the miR-27b binding site or the mutant site are shown in Figure 3A. In HEK293 cells, co-transfection of the wild-type (WT) 3’-UTR with miR-27b MM significantly reduced the relative luciferase activity (p<0.001), and there was no significant change of the relative luciferase activity after co-transfection of mutant-type (MUT) 3’-UTR with miR-27b MM (p>0.05) (Figure 3B). Moreover, in PC12 cells, transfection of miR-27b MM significantly suppressed the firefly luciferase reporter activity of the WT 3’-UTR (p<0.01), but not that of the MUT 3’-UTR (p>0.05) (Figure 3C).

### ICV injection of miR-27b AM promoted the ICH-induced Nrf2/ARE pathway activation in the experimental ICH rat model

**Figure 4:**
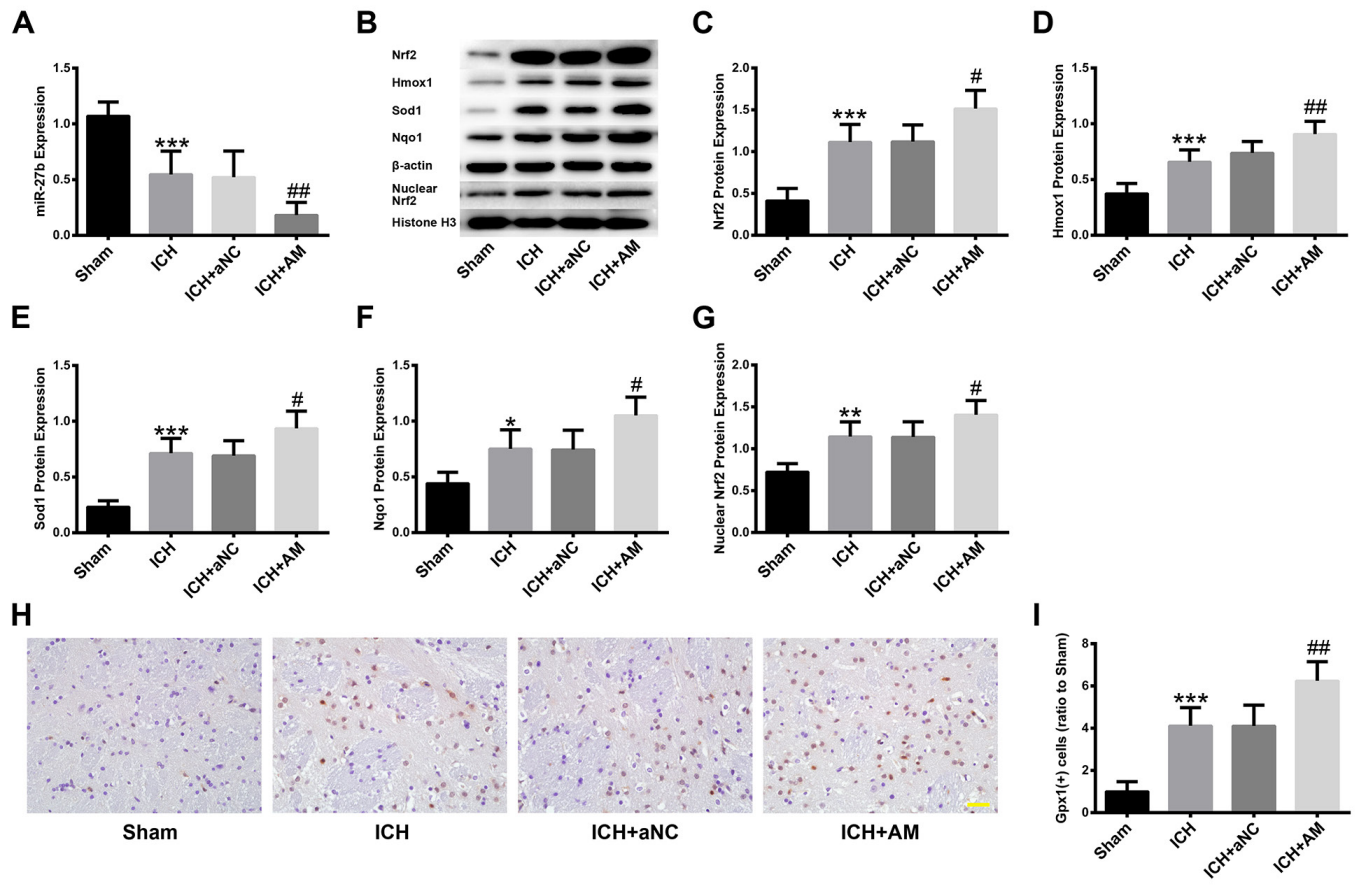
ICV injection of miR-27b AM promoted the ICH-induced Nrf2/ARE pathway activation in rats ICV injection of miR-27b AM was performed at 3 d before ICH and rats were sacrificed for qRT-PCR, Western blot and IHC at 1 d after ICH. **(A)** Analysis of qRT-PCR data for miR-27b. **(B)** Immunoblots of Nrf2, Hmox1, Sod1, Nqo1 and nuclear Nrf2. Immunoblotting analysis of **(C)** Nrf2, **(D)** Hmox1, **(E)** Sod1, **(F)** Nqo1 and **(G)** nuclear Nrf2. **(H)** IHC images of Gpx1 in rat striatum. Scale bar=20 μm. **(I)** Bar graphs showing quantification of Gpx1 positive cells and values are expressed relative to the sham controls. Data are presented as the mean ± SD (n=6). *p<0.05, **p<0.01, ***p<0.001 vs Sham, #p<0.05, ##p<0.01 vs ICH.

To investigate the protective effects of miR-27b inhibition on ICH, ICV injection of miR-27b AM was performed. The efficiency of AM and its effects on Nrf2 expression were detected at 3 d post-injection (Supplementary Figure 2). Subsequently, rats were subjected to ICH and sacrificed at 1 d later. The results indicated that miR-27 AM further promoted the decrease in miR-27b level caused by ICH (p<0.01), as shown in Figure 4A. Compared with the Sham group, the protein levels of Nrf2, Hmox1, Sod1, Nqo1 and nuclear Nrf2 after ICH were significantly upregulated (all p<0.05), and AM further promoted those effects (all p<0.05) (Figure 4B–4G). Immunohistochemistry (IHC) analysis was used to detect another Nrf2-regulated enzyme, namely Gpx1, and the result revealed that there were more Gpx1-positive cells in the ICH group than in the Sham group (p<0.001). Moreover, compared with the ICH group, miR-27b AM injection significantly increased the number of Gpx1-positve cells (p<0.01) (Figure 4H, 4I).

### ICV injection of miR-27b AM attenuated the ICH-induced oxidative damage and neuroinflammation in the experimental ICH rat model

**Figure 5:**
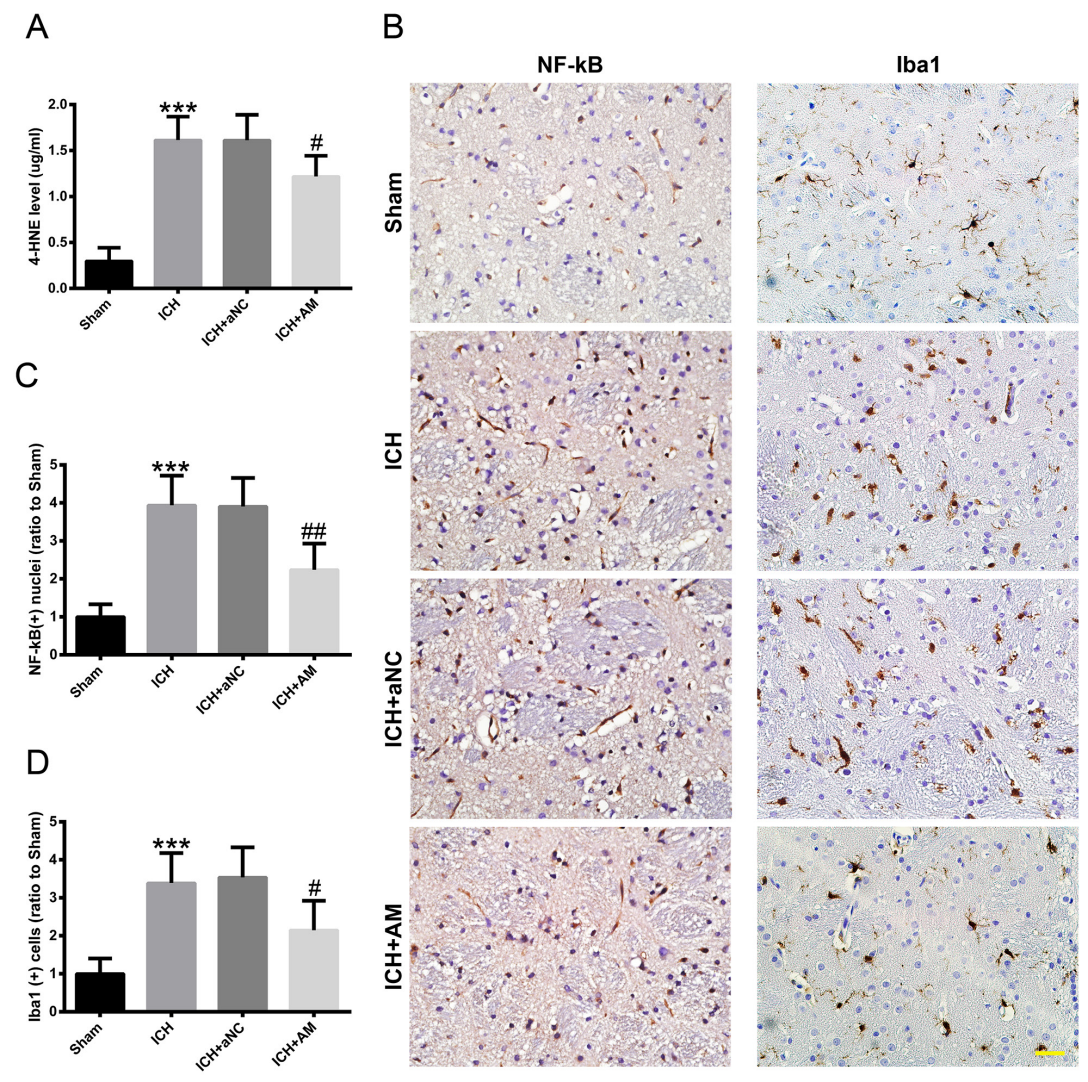
ICV injection of miR-27b AM attenuated ICH-induced oxidative damage and neuroinflammation in rats 3 d after ICV injection, rats were subjected to experimental ICH, and then they were sacrificed for ELISA and IHC at 1 day later. **(A)** 4-HNE level in rat striatum. **(B)** IHC images of NF-κB and Iba1 in rat striatum. Scale bar=20 μm. Bar graphs showing quantification of nuclear-positive cells of **(C)** NF-κB and **(D)** Iba1 positive cells, and the values are expressed relative to the sham controls. Data are presented as the mean ± SD (n=6). ***p<0.01 vs Sham, #p<0.05, ##p<0.01 vs ICH.

ICH increased the 4-hydroxynonenal (4-HNE) level (p<0.001), whereas miR-27b AM injection diminished the ICH-induced 4-HNE increase (p<0.05) (Figure 5A). In the sham group, NF-κB was mainly located in the cytoplasm and the nuclear-positive cells were rare (Figure 5B), whereas in the ICH group, NF-κB was mainly located in the nuclei and the number of nuclear-positive cells was significantly increased (p<0.001). In the ICH+AM group, NF-κB was also mainly located in the nuclei, but the number of nuclear-positive cells was significantly decreased (p<0.01) (Figure 5B, 5C). To detect microglia in rat striatum, ionized calcium-binding adapter molecule 1 (Iba1) was used in IHC analysis. In the sham group, Iba1-positive cells included numerous branching processes and had little cytoplasm, whereas in the ICH group the positive cells were devoid of branching processes and exhibited enlarged cytoplasm and cell bodies (cell body diameter: 3.806 ± 0.062 μm vs. 3.052 ± 0.059 μm, p<0.001), irregular shapes, consistent with the morphological characteristics of activated microglia (Figure 5B). Additionally, the number of Iba1-positive cells in the ICH group was significantly higher than that in the Sham group (p<0.001) (Figure 5D). In the ICH+AM group, the Iba1-positive cells were still in an activated state, but their number was significantly reduced (p<0.05) (Figure 5B, 5D).

### ICV injection of miR-27b AM decreased cell death and neurological deficits but not lesion volume in the experimental ICH rat model

**Figure 6:**
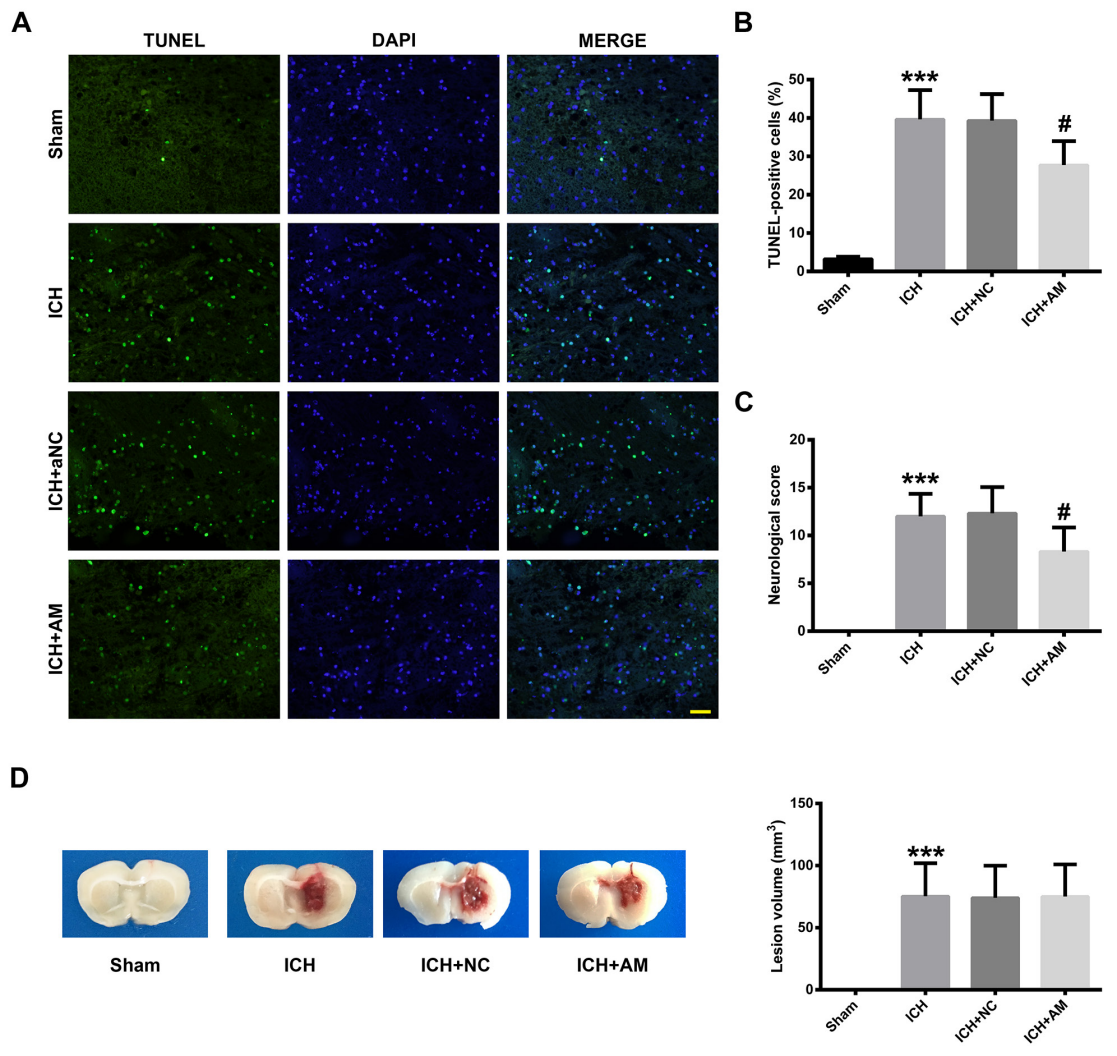
ICV injection of miR-27b AM alleviated cell death, neurological deficits but not lesion volume in ICH rats **(A)** TUNEL staining (green) of perihematomal tissue at 1 day after ICH. Nuclei were counterstained with DAPI (blue). Scale bar=20 μm. **(B)** Bar graphs showing quantification of TUNEL positive cells and the values are shown as the percentage relative to the counts of DAPI-stained cells. **(C)** Bar graphs showing the neurological deficits which were assessed with a 24-point neurological scoring system at 1 d after ICH. **(D)** Representative coronal sections (2 mm thickness) of the rat brain in four groups. **(E)** Quantitative analysis of the lesion volume on day 1 post-ICH. Data are presented as the mean ± SD (n=6). ***p<0.001 vs Sham, #p<0.05 vs ICH.

TUNEL staining revealed a high density of positively-stained cells within the hemorrhagic lesion (Figure 6A). Quantitative analysis showed that ICH increased the rate of TUNEL-positive cells (p<0.001), whereas miR-27b AM injection diminished the ICH-induced cell death (p<0.05) (Figure 6B). To further investigate the effects of miR-27b AM on neurologic function in ICH rats, assessment of neurobehavioral deficits was performed at 1 d after ICH. Compared with the rats in the Sham group, the ICH rats showed varying degrees of neurological damage symptoms (p<0.001). However, ICV injection of miR-27b AM significantly reduced the neurological deficits caused by ICH (p<0.05) (Figure 6C). Lesion volume was measured by morphometric measurement (image analysis) at 1 d post-ICH. As shown in Figure 6D, no detectable bleeding was observed in sham-operated rats, and collagenase injection could lead to significant brain injury. The lesion volume for the ICH and ICH+AM groups were 75.36 ± 10.87 mm^3^ vs. 75.12 ± 10.54 mm^3^, thus, there was no significant difference between the groups (p>0.05), indicating that miR-27b AM did not affect the lesion volume (Figure 6E).

### MiR-27b IN transfection attenuated the ferrous sulfate-induced cell injury and apoptosis, OS and the inflammatory response in PC12 cells via the Nrf2/ARE pathway

**Figure 7:**
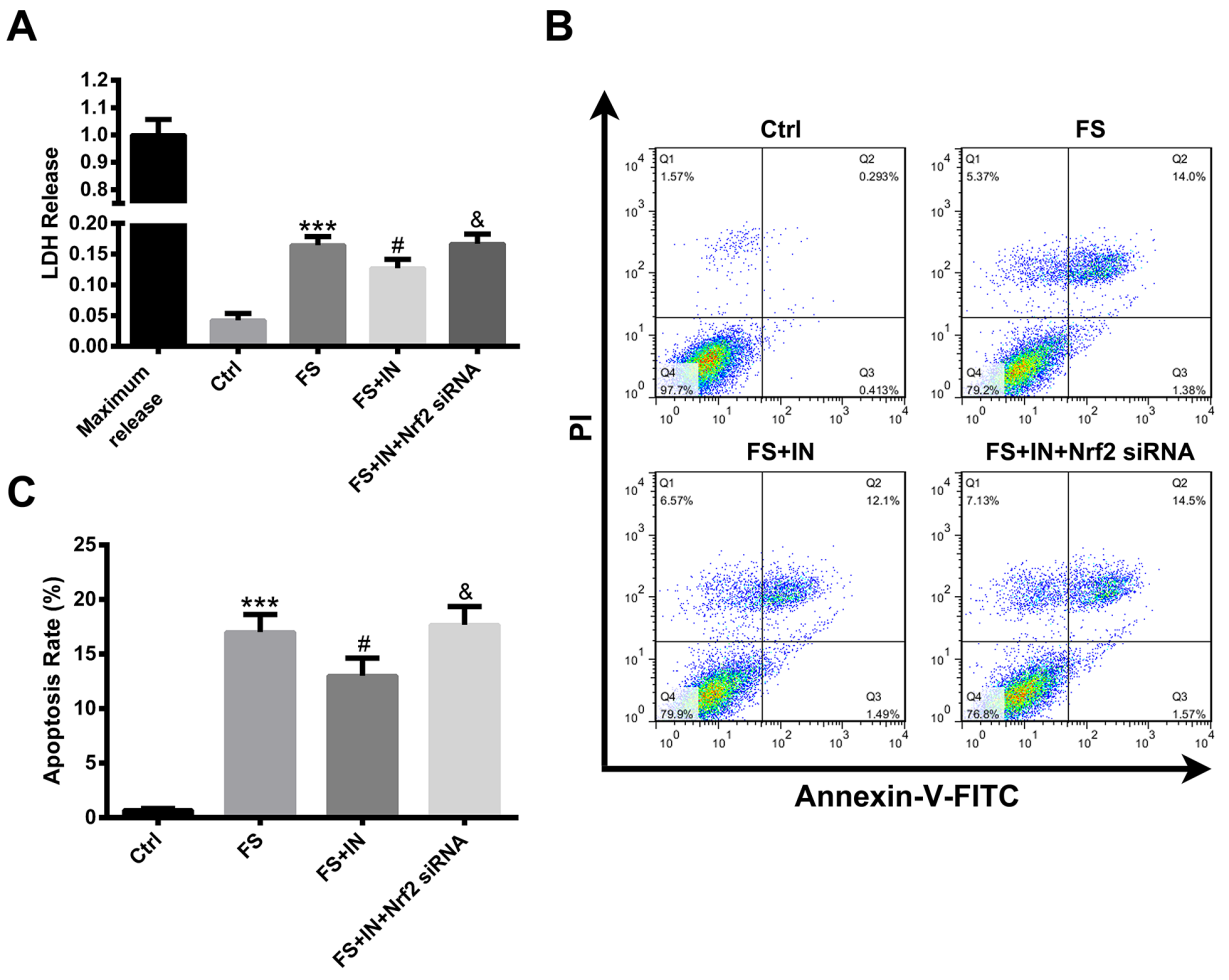
MiR-27b IN transfection attenuated the FS-induced cell injury and apoptosis in PC12 cells **(A)** LDH levels in culture medium. **(B)** Flow cytometric analysis of apoptotic cells. **(C)** Quantification of apoptotic cells (AV+/PI+, late-phase apoptotic cells; AV+/PI−, early-phase apoptotic cells). Data are presented as mean ± SD (n=3). ***p<0.001 vs Ctrl, #p<0.05 vs FS, &p<0.05 vs FS+IN.

To confirm that miR-27b inhibition attenuated the iron-induced cell injury and apoptosis in neural cells, we transfected PC12 cells with miR-27b IN before ferrous sulfate (FS) stimulation, and then cell injury and apoptosis were evaluated by lactate dehydrogenase (LDH) release and flow cytometry, respectively. The results revealed that the treatment with miR-27b IN significantly attenuated the FS-induced LDH release and cell apoptosis (both p<0.05), as shown in Figure 7. To demonstrate that the protective effects of miR-27b inhibition on FS stimulation were dependent on the Nrf2/ARE pathway, we co-transfected Nrf2 siRNA with miR-27b IN into PC12 cells. The efficiency of gene knockdown was confirmed by assaying the Nrf2 protein level (Supplementary Figure 3C). Suppression of Nrf2 could block the protective effects of miR-27b inhibition on cell injury and apoptosis (both p<0.05).

**Figure 8:**
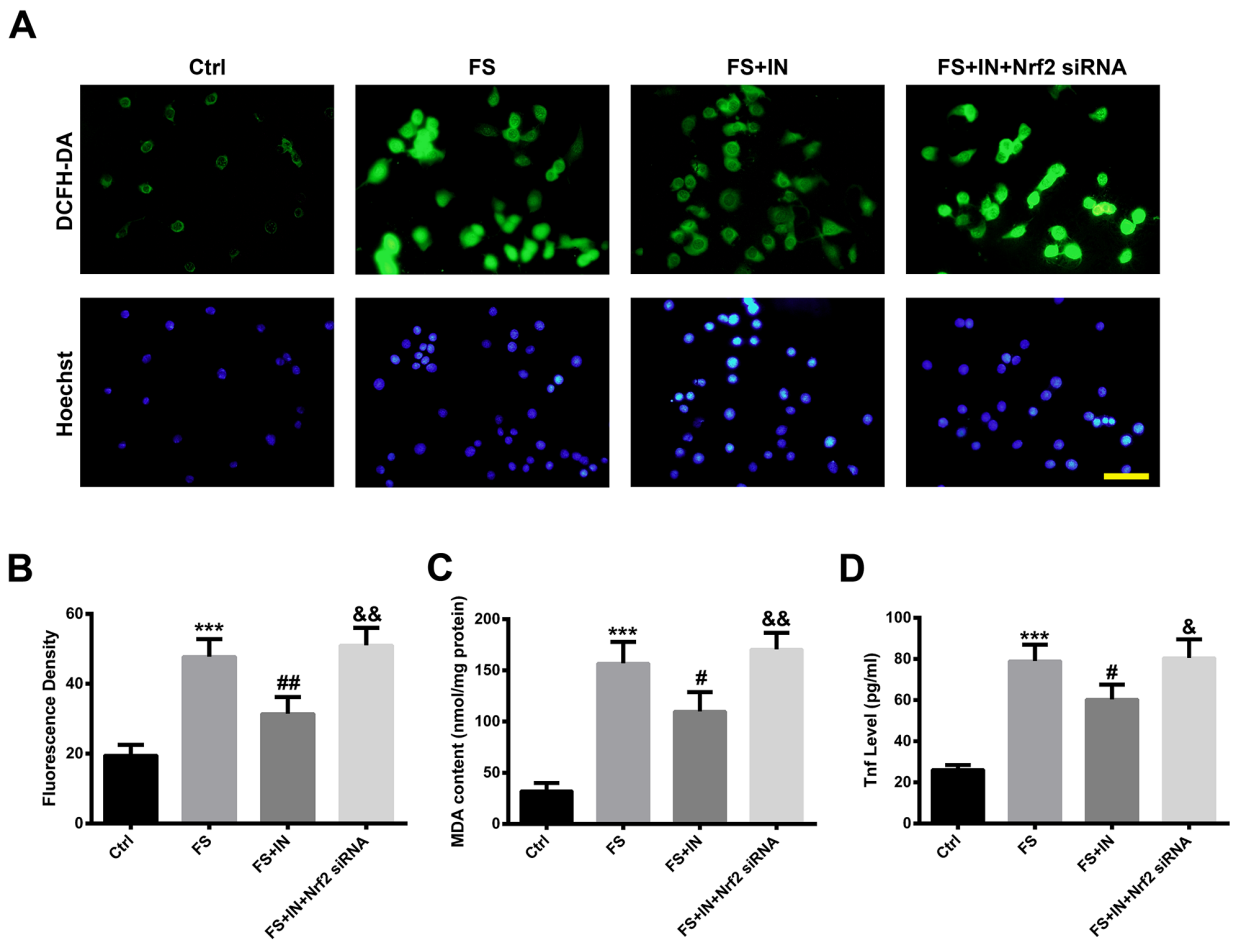
MiR-27b IN transfection attenuated the FS-induced OS and inflammatory response in PC12 cells **(A)** Detection of ROS using DCFH-DA (green) by fluorescence microscopy. Nuclei were counterstained with Hoechst 33342 (blue). Scale bar = 20 μm. **(B)** Quantification of fluorescence density. **(C)** MDA content in PC12 cells. **(D)** Tnf level in culture supernatants. Data are presented as the mean ± SD (n=3). ***p<0.001 vs Ctrl, #p<0.05, ##p<0.01 vs FS, &p<0.05, &&p<0.01 vs FS+IN.

To further investigate whether the inhibition of miR-27b increased the anti-oxidative and anti-inflammatory ability of neural cells, reactive oxygen species (ROS) production, malondialdehyde (MDA) content and Tnf expression in PC12 cells were assessed. As shown in Figure 8A, intracellular ROS evaluation by 2′, 7′-dichlorofluorescin diacetate (DCFH-DA) revealed that in the control group, cells exhibited weak green fluorescence, whereas in the FS group, cells had higher fluorescence density (p<0.001). The elevated ROS production was reduced by transfection of miR-27 IN (p<0.01) and the effect was blocked by Nrf2 knockdown (p<0.01) (Figure 8B). In addition, the increased levels of MDA and Tnf after FS treatment were diminished by miR-27b IN (both p<0.05), and those effects were inhibited by Nrf2 knockdown (both p<0.05) (Figure 8C, 8D).

## DISCUSSION

In this study, we investigated the protective effects of miR-27b inhibition on ICH rats and iron-exposed PC12 cells. Following ICH in rats, striatal expression of miR-27b was decreased in a time-dependent manner and was negatively correlated with Nrf2 expression. MiR-27b modulates Nrf2 expression by directly targeting the Nrf2 mRNA. ICV injection of miR-27b AM promoted Nrf2 signaling in ICH rats, and reduced cerebral OS, inflammation, cell death and neurological deficits. Additional studies revealed that the mechanism of miR-27b inhibition in ICH might involve the Nrf2/ARE defense pathway. Indeed, our data demonstrated that miR-27b inhibition protected against cerebral ICH injury by stimulating the anti-oxidative and anti-inflammatory responses in neural cells, offering a novel therapeutic strategy for ICH.

Recent evidence suggests that OS modulates miR biogenesis by affecting key molecules involved in miR maturation, such as the nuclear export factor exportin 5 and Dicer [34, 35]. In addition, miRs themselves can be modified by OS, which alters their integrity, stability, binding affinity and function [36]. Furthermore, several miRs have been shown to be involved in the cellular response to OS via the anti-oxidative pathways [37, 38]. In fact, miR-27b, an OS-responsive neuronal miR, is reportedly downregulated after oxidative stimulation [18–20]. Consistent with these findings, we observed that miR-27b expression was reduced at the early stage of ICH, a period with significant oxidative damage [33]. Nrf2, a key regulator that maintains the intracellular redox homeostasis, was identified as a potential target of miR-27b by miRNA target prediction tools. A time-course study performed in ICH model rats showed that downregulation of miR-27b was consistent with the upregulation of the Nrf2 mRNA and protein. Further correlational analysis confirmed the negative correlation between the expression of miR-27b and the Nrf2 protein. In view of the neuroprotective effects of Nrf2 in the ICH model and the potential interaction between Nrf2 mRNA and miR-27b, the down-regulation of miR-27b could be regarded as a protective response to ICH.

MiR-27b overexpression decreased the Nrf2 mRNA level, and miR-27b inhibition exerted the opposite effect. To examine the mechanism responsible for these effects, we performed bioinformatics search for potential miR-27b binding sites within the 3’-UTR of the Nrf2 mRNA. Moreover, dual luciferase assays showed that Nrf2 mRNA was a direct target of miR-27b. These results indicated that miR-27b modulates Nrf2 expression at the posttranscriptional level by combining with the 3’-UTR of Nrf2 mRNA and promoting its degradation. In addition, the expression of nuclear Nrf2, Hmox1, Sod1 and Nqo1 was also regulated by miR-27b modulation, suggesting that miR-27b regulates the Nrf2/ARE pathway by controlling Nrf2 expression [27]. The regulatory effects of miR-27b on Nrf2 suggest that the increased Nrf2 expression after ICH was due, at least in part, to the downregulation of miR-27b. The miR-27b downregulation might play a critical role in the protective responses to ICH, indicating the potential of miR-27b inhibition as a neuroprotective strategy for ICH via the promotion of the Nrf2/ARE pathway.

To investigate the protective effects of miR-27b inhibition on ICH-induced brain injury, ICV injection of miR-27b AM was performed, followed by ICH induction. MiR-27b AM further reduced the miR-27b expression and promoted the ICH-induced Nrf2/ARE pathway activation, as evidenced by the further increase in the expression of nuclear Nrf2, Hmox1, Sod1, Nqo1 and Gpx1. OS and neuroinflammation are thought to be the main factors involved in the progression of ICH-induced secondary brain injury [4]. We specifically tested the protective effects of miR-27b inhibition at 1 d after ICH because half of the deaths due to ICH occur within the first 2 days [39]. Besides, in other studies the ICH-mediated damage was also evaluated at 1 d after ICH [33, 40]. In this study, ICH increased the level of 4-HNE, a natural byproduct of lipid peroxidation, which is often used as a marker of OS [32]. It also promoted nuclear translocation of NF-κB, which induced the transcription of several important pro-inflammatory genes [41]. Additionally, ICH increased the number and intensity of activated microglia in the perihematomal area, which constituted a source of ROS, cytokines, nitric oxide and other potentially toxic factors after ICH [4, 9]. ICV injection of miR-27b AM could attenuate the ICH-induced lipid peroxidation and the activation of NF-κB and microglia. Furthermore, miR-27b AM effectively reduced cell death after ICH. Even though miR-27b AM did not decrease the lesion volume, it could effectively reduce the neurological deficits after ICH, which might be attributed to the attenuation in neuronal damage [33]. In this study, the protective effects of AM were accompanied by the increased expression of Hmox1. Although some available evidence also indicated that Hmox1 exerted cytoprotective effects [42, 43], previous research found that Hmox1 knock-out (KO) mice had smaller injury volume than the wild-type mice after ICH [44]. Besides, it was previously reported that Hmox1 might be induced through an Nrf2-independent pathway [39]. Therefore, additional efforts should be made to clarify the complex function and regulatory mechanism of Hmox1 in ICH.

The Nrf2/ARE pathway is an important cellular defense mechanism against ICH-induced cerebral injury [32, 33]. To directly investigate the role of the Nrf2/ARE pathway in the miR-27b inhibition-mediated neuroprotection, an *in vitro* iron toxicity model with PC12 cells was utilized. Free iron promotes ROS formation via the Fenton reaction and, in turn, induces oxidative injury [45]. In this study, cell injury was evaluated by detecting the LDH level in the culture medium, which is known to be correlated with the number of damaged cells [46]. MiR-27b IN transfection reduced the LDH release and cell apoptosis after FS stimulation in PC12 cells, suggesting that miR-27b inhibition protects PC12 cells from the neurotoxicity of iron. Moreover, miR-27b IN transfection attenuated the FS-induced increase in ROS production, Tnf expression and MDA content, which is another OS marker that directly reflects the extent of lipid peroxidation [47]. Nrf2 knockdown blocked the protective effects of miR-27b inhibition, strongly suggesting the Nrf2/ARE pathway is essential for the protective effects of miR-27b inhibition on the iron-induced oxidative and inflammatory damage to PC12 cells. However, in order to determine whether the protective effects of miR-27b inhibition are Nrf2-dependent in vivo, studies using Nrf2 KO mice are still needed.

Our study has several limitations. Although the iron toxicity model in PC12 cells has already been used to study the iron-induced brain injury after ICH [48], it is actually impossible to simulate the complicated mechanisms involved in the post-hemorrhagic neuronal damage in clinical patients, such as the mass effects or tissue hypoperfusion. In addition, the miR expression and protective effects of miR-27b inhibition derived from *in vitro* FS injury may differ from the brain tissue response to ICH. The therapeutic application of miR-27b AM for ICH is probably barred by the need for pre-treatment, tissue-specific delivery and avoidance of degradation. Alternate routes for AM delivery, such as intranasal, as considered for drug delivery in several neurological diseases [49–52], could avoid blood brain barrier exclusion of AM *in vivo* [53], and facilitate its translation to the clinic for the treatment of ICH.

In summary, miR-27b levels were reduced after ICH, which contributed to an increase in Nrf2 expression. MiR-27b inhibition diminished ICH-induced oxidative and inflammatory injury, raising the prospect of using miR-27b inhibition as a therapeutic strategy for ICH.

## MATERIALS AND METHODS

### ICH model

Male Wistar rats were provided by the Laboratory Animal Center of Shandong University. All animal studies conformed to the guidelines outlined in the *Guide for the Care and Use of Laboratory Animals* from the National Institutes of Health and were approved by the ethics committee of Qilu Hospital. Experimental ICH was induced by intracerebral injection of collagenase as previously described [54, 55]. Briefly, rats were placed on a stereotaxic apparatus (Zhongshi Dichuang, Beijing, China) after anesthesia. An incision was made in the middle of the scalp and a burr hole was drilled with a dental drill. Then a microsyringe (Gaoge, Shanghai, China) was stereotaxically implanted through the hole into the right striatum (coordinates: 0.2 mm posterior to bregma, 6.0 mm below the skull, and 3.0 mm right to the middle line). ICH was induced by administration of 1 μl of saline containing 0.23 U bacterial collagenase type IV (Solarbio, Beijing, China) over a 5-min period. To avoid backflow, the microsyringe was kept in situ for another 10 min before being slowly withdrawn. After collagenase infusion, craniotomies were sealed with bone wax, and wounds were sutured. Sham operation was performed with the stereotaxic injection of an equal volume (1 μl) of saline instead of collagenase.

### Animal experimental protocol

ICV injection of miR-27b AM (RiboBio, Guangzhou, China) was performed to inhibit the endogenous miR-27b in vivo. MiR-27b AM and the AM negative control (aNC) were dissolved in sterile normal saline to a final concentration of 20 nmol/L [56]. A total of 5 μl of the mixture or normal saline was slowly injected into the right lateral ventricle according to the following coordinates: 1.5 mm posterior to the bregma, 1.1 mm right to the middle line, 4.5 mm deep from the surface of the skull [57].The inhibitory efficiency of AM was evaluated at 3 d after the ICV injection. The sequences of miR-27b AM and aNC are shown in Table 1.

**Table 1:** Sequences of miR-27b antagomir (AM) and antagomir-negative control (aNC) used for ICV injection

miR-27b AM	mGmCmAmGmAmAmCmUmUmAmGmCmCmAmCmUmGmUmGmAmA
aNC	mCmAmGmUmAmCmUmUmUmUmGmUmGmUmAmGmUmAmCmAmA

In group 1 (Sham group), an ICV injection of 5 μL normal saline was administered at 3 d prior to the Sham operation. In group 2 (ICH group), an ICV injection of 5 μL normal saline was given at 3 d before ICH. In group 3, (ICH+aNC group) an ICV injection of 5 μL aNC was administered at 3 d prior to ICH. In group 4, (ICH+AM group) an ICV injection of 5 μL AM was performed at 3 d before ICH. Rats were sacrificed for further analysis at 1 d after ICH.

### Neurological deficits

All the rats were scored blindly for neurological deficits using a 24-point neurological scoring system at 1 d after ICH [33]. The tests included body symmetry, gait, climbing, circling behavior, front limb symmetry and compulsory circling. Each test was graded from 0 to 4, establishing a maximum deficit score of 24. Immediately after the tests, rats were sacrificed for lesion volume evaluation.

### Lesion volume analysis

Rat brains were cut coronally through the needle entry site (identifiable on the brain surface) to obtain serial slices (2-mm thickness) anterior and posterior to the needle entry plane. Digital photography of the serial slices was taken and the lesion area was measured using ImageJ 1.44 (US National Institutes of Health, Bethesda, MD, USA). The total lesion volume (mm^3^) was calculated by summing the lesion area in each section and multiplying by the distance between sections [58].

### Tissue preparation

For IHC analysis and TUNEL staining, rats were perfused through the heart with cold saline followed by 4% paraformaldehyde. Brains were carefully removed and fixed in 4% paraformaldehyde at 4°C for 24 hours. After fixation, they were paraffin-embedded and coronally processed into 5 μm sections. For qRT-PCR, Western blot analysis and enzyme-linked immunosorbent assay (ELISA), rats were perfused with cold saline. Brains were then dissected on ice immediately to obtain the ipsilateral striatum, and the tissue were flash-frozen in liquid nitrogen and stored at -80°C for further use.

### qRT-PCR

Total RNA from rat striatum and PC12 cells was isolated using TRIzol reagent (Invitrogen, Carlsbad, CA, USA) according to the manufacturer’s protocol. PrimeScript™ RT reagent Kit with gDNA Eraser (Takara, Kusatsu, Japan) and Mir-X™ miRNA First-Strand Synthesis (Takara) were used to synthesize cDNA from mRNA and miR, respectively. qRT-PCR was performed using the iQ5 Real Time PCR System (Bio-Rad, Hercules, CA, USA) with the SYBR Premix Ex Taq™ (Takara). All measurements were performed in triplicate. Data were analyzed using the 2^-ΔΔCT^ method [59]. U6 and β-actin were used to normalize the miRNA and mRNA level, respectively. Common downstream primer for miR qRT-PCR is supplied with the kit. All the other primers for qRT-PCR were synthesized by Gene Pharma (Shanghai, China) and the sequences are shown in Table 2.

**Table 2:** Primers used for qRT-PCR

Gene name	Forward (5’ to 3’)	Reverse (5’ to 3’)
**miR-27b**	**TTCACAGTGGCTAAGTTCTGC**	
**U6**	**CTCGCTTCGGCAGCACA**	**AACGCTTCACGAATTTGCGT**
**Nrf2**	**TTTGTAGATGACCATGAGTCGC**	**TGTCCTGCTGTATGCTGCTT**
**β-actin**	**AGACCTTCAACACCCCAG**	**CACGATTTCCCTCTCAGC**

### Protein extraction and western blot

Protein extractions from rat striatum and PC12 cells were analyzed. Total protein extraction was performed using the RIPA lysis buffer (Beyotime, Nantong, China), and the nuclear protein was isolated using the nuclear and cytoplasmic protein extraction kit (Beyotime) according to the manufacturer’s instructions. Protein concentration was measured using the bicinchoninic acid (BCA) kit (Beyotime). Equal amounts of protein were separated by sodium dodecyl sulfate–polyacrylamide gel electrophoresis and transferred to polyvinylidene fluoride membranes (Millipore, Billerica, MA, USA). The membranes were blocked for 1 h in 5% non-fat milk and then incubated overnight at 4°C with the primary antibodies against Nrf2 (1:1000; Abcam, Cambridge, UK), Hmox1 (1:10000; Abcam) Sod1 (1:500; Proteintech, Wuhan, China), Nqo1 (1:1000; Abcam,) and Histone H3 (1:500; Proteintech), β-actin [1:1000; Zhongshan Golden Bridge (ZGBB), Beijing, China]. After incubation, the membranes were washed in 1×TBST (50 mM Tris-HCl, pH 7.4, 150 mM NaCl with 0.1% Tween), and then incubated with horseradish peroxidase labeled secondary antibodies (1:5000; ZGBB) at room temperature for 2 h. After washing with 1×TBST, membranes were visualized with an Amersham Imager 600 (GE, Boston, MA, USA) and Immobilon Western Chemiluminescent HRP Substrate kit (Millipore). Immunoreactive labeling was analyzed with the ImageJ software and standardized against the protein level of β-actin or Histone H3.

## IHC

Brain sections were deparaffinized in xylene and then rehydrated through graded ethanol washes. Antigen retrieval was performed with 10 mM sodium citrate followed by 20 min incubation in H_2_O_2_ to quench the endogenous peroxidase. After that, sections were blocked with 10% goat serum, and then incubated overnight with primary antibodies against Gpx1 (1:300; Abcam), NF-κB (1:1000; Abcam) and Iba1 (1:1000; Wako Chemicals, Richmond, VA). After rinsing with PBS, sections were incubated with a biotinylated secondary antibody (ZGBB) followed by an avidin-biotin horseradish peroxidase complex (ZGBB). The color reaction was developed by incubation with diaminobenzidine (ZGBB) and nuclei were counterstained with hematoxylin. Slides were dehydrated in graded alcohol and xylene, and then mounted with coverslips. Coronal sections at the level of the needle insertion (collagenase injection site) were chosen in quantification analysis. Five fields adjacent to the hematoma on each slide were randomly imaged using an Olympus microscope (DP72, Tokyo, Japan). Tissue samples from 6 rats per each group were prepared for IHC. The number of positively stained cells was counted using the Image-pro plus 6.0 (Media Cybernetics Inc., Rockville, MD, USA) and the mean per section was calculated. The final data are reported relative to sham controls.

## ELISA

4-HNE content in rat striatum and Tnf expression in culture medium were measured using the OxiSelect™ HNE Adduct ELISA kit (Cell BioLabs, San Diego, CA, USA) and Platinum ELISA kit (eBioscience, Hatfield, UK) according to the manufacturers’ instructions, respectively. Optical density (OD) values were measured using the Varioskan Flash spectral scanning multimode reader (Thermo Electron, Vantaa, Finland).

### TUNEL staining

To assess cell death in vivo, TUNEL staining was performed using the in situ Cell Death Detection Kit, POD (Roche, Mannheim, Germany) according to the manufacturer’s instructions, and the nuclei were counter-stained with 4’, 6-diamidino-2-phenylindole dihydrochloride (DAPI; Solarbio). Fluorescence was visualized with an Olympus microscope (DP72). Coronal sections at the level of the needle insertion were used in the quantification analysis. Five fields adjacent to the hematoma on each slide were randomly imaged and tissue samples from 6 rats for each group were prepared for TUNEL straining. The number of TUNEL/DAPI positive cells was counted using the Image-pro plus 6.0 and the mean per section was calculated.

### Cell culture and treatment

HEK293 cells obtained from American Type Culture Collection (ATCC, Manassas, VA, USA) were cultured in DMEM (Gibco-Thermo-Fisher, Waltham, MA, USA) supplemented with 10% (v/v) fetal bovine serum (FBS) and kept at 37°C in a humidified 5% CO_2_ atmosphere.

We used the iron toxicity model in PC12 cells to examine the effects of iron on neural cells *in vitro* [48]. PC12 cells obtained from the Cell Bank of the Chinese Academy of Sciences (Shanghai, China) were cultured in DMEM containing 5% FBS (Gibco-Thermo-Fisher) and 10% horse serum (Gibco-Thermo-Fisher) and maintained at 37°C in a humidified 5% CO_2_ atmosphere. MiR-27b MM, mNC, IN, iNC and Nrf2 siRNA (all purchased from Gene Pharma, Shanghai, China) were transfected into PC12 cells for 36 h using INTERFERin™ (Polyplus, Illkirch, France) following the manufacturer’s instruction. The miR oligo sequences are listed in Table 3. To develop the iron toxicity model in vitro, PC12 cells were stimulated with 10 μM FS (Sigma, St. Louis, MO, USA) for 1 d according to a previous study [46].

**Table 3:** Sequences of miR oligo used for transfection

miR oligo	Sequence (5’-3’)
miR-27b mimics	UUCACAGUGGCUAAGUUCUGC
	AGAACUUAGCCACUGUGAAUU
mimics-negative control	UUCUCCGAACGUGUCACGUTT
	ACGUGACACGUUCGGAGAATT
miR-27b inhibitor	GCAGAACUUAGCCACUGUGAA
Inhibitor-negative control	CAGUACUUUUGUGUAGUACAA

### Dual-luciferase reporter assay

The WT Nrf2 3’-UTR (444-bp) (Supplementary Table 1) containing the binding site for miR-27b or a MUT Nrf2 3’-UTR (444-bp) (Supplementary Table 1) were amplified and inserted into the pmirGLO vector (Promega, Madison, WI, USA) with XhoI and SacI double digestion. Both recombinant vectors were verified by DNA sequencing. The HEK293 cells and PC12 cells subcultured in 96-well plates were cotransfected with either of the recombinant vectors and miR-27b MM or mNC using Lipofectamine 2000 (Invitrogen). Cells were lysed 48 hours after transfection and subjected to a double luciferase reporter assay system (Promega) using the Varioskan Flash spectral scanning multimode reader. Renilla luciferase activity was normalized to that of firefly luciferase.

### Immunofluorescence

After 36 h post-transfection with miR-27b MM or IN, PC12 cells were fixed with 4% paraformaldehyde for 20 min and blocked with 10% goat serum for 30 min. The glass slides with cells were then incubated at 4°C overnight with the primary antibody against Nrf2 (1:200; Abcam). After rinsing three times, slides were incubated with the FITC-conjugated secondary antibody (1:200, ZGBB) for 1 h. The nuclei were counterstained with DAPI. Images were captured with an Olympus microscope (DP72). The fluorescence density was analyzed in at least 3 separate experiments using the Image-pro plus 6.0.

### Measurement of intracellular ROS

Intracellular ROS level was measured using DCFH-DA (Sigma). After FS treatment for 1 h, PC12 cells were rinsed and incubated with 10 μM DCFH-DA in DMEM for 30 min at 37 °C under dark conditions. Nuclei were counterstained with Hoechst 33342 (Solarbio). After that, cells were washed 3 times and visualized with an Olympus microscope (DP72). As previously reported, the mean fluorescence density, used as an index of the ROS production, from 5 random fields was measured with the Image-pro plus 6.0 [60]. The experiment was carried out in triplicate.

### LDH assay

One day after the FS treatment, the LDH activity in the culture medium of PC12 cells was detected using a LDH assay kit (Beyotime) according to the manufacturer’s instruction. OD values were measured at 490 nm using the Varioskan Flash spectral scanning multimode reader. The LDH release was calculated using the following formula: [OD_sample_ − OD_blank_]/[OD_maximumrelease_ − OD_blank_].

### Flow cytometry

One day after FS treatment, PC12 cells were collected, and cell apoptosis was evaluated by flow cytometry using an Annexin-V-FITC/PI kit (BD, Franklin Lakes, NJ, USA) according to the manufacturer’s instruction. The data was analyzed by the software of Flowjo 7.6 (Ashland, OR, USA).

### MDA measurement

One day after FS treatment, PC12 cells were lysed with cell lysis buffer, and the protein concentration in the supernatant was determined using the BCA kit. The MDA content was assayed with a Lipid Peroxidation MDA Assay Kit (Beyotime) following the manufacturer’s instructions. MDA levels were normalized to milligram of total protein.

### Statistical analysis

Data were expressed as means ± SD of at least three independent experiments and were analyzed by Prism software 5.0 (GraphPad, Inc., La Jolla, CA, USA). Statistical analyses were conducted using Student’s t-test or one-way ANOVA with the post hoc Tukey’s test. Pearson correlation analysis was used to analyze the relation between miR-27b and Nrf2 protein, and Student’s t-test was used for the hypothesis testing of Pearson correlation coefficient (r). A value of P<0.05 was the criterion for significance.

## SUPPLEMENTARY MATERIALS FIGURES AND TABLE



## Abbreviations

OS: oxidative stress
ICH: intracerebral hemorrhage
miR-27b: microRNA-27b
AM: antagomir
IN: inhibitor
NF-κB: nuclear factor-κB;
3’-UTR: 3’-untranslated region
t-BHP: tert-Butyl hydroperoxide
Nrf2: nuclear factor erythroid 2-related factor 2
Keap1: kelch-like ECH-associated protein-1
ARE: antioxidant response element
Hmox1: heme oxygenase 1
Sod1: superoxide dismutase 1
GSTs: glutathione transferases
Gpx1: glutathione peroxidase 1
NADPH: nicotinamide adenine dinucleotide phosphate
Nqo1: NADPH: quinone oxidoreductase-1
ICV: intracerebroventricular
qRT-PCR: quantitative real-time polymerase chain reaction
MM: mimics
mNC: MM negative control
iNC: IN negative control
WT: wild-type
MUT: mutant-type
IHC: immunohistochemistry
4-HNE: 4-hydroxynonenal
Iba1: ionized calcium-binding adapter molecule 1
FS: ferrous sulfate
LDH:|: lactate dehydrogenase
ROS: reactive oxygen species
MDA: malondialdehyde
DCFH-DA: 2’, 7’-dichlorofluorescin diacetate
aNC: AM negative control
ELISA: enzyme-linked immunosorbent assay
BCA: bicinchoninic acid
OD: optical density
DAPI: 4’, 6-diamidino-2-phenylindole dihydrochloride
FBS: fetal bovine serum
