# Supplementary material for: MicroRNA-27b inhibition promotes Nrf2/ARE pathway activation and alleviates intracerebral hemorrhage-induced brain injury
Source: Oncotarget. 2017 Aug 7;8(41):70669–84. doi: 10.18632/oncotarget.19974 (PMC5642585; doi:10.18632/oncotarget.19974)
Supplement: Supplementary file 1 [file oncotarget-08-70669-s001.pdf]

MicroRNA-27b inhibition promotes Nrf2/ARE pathway activation and alleviates intracerebral hemorrhage-induced brain injury

SUPPLEMENTARY MATERIALS

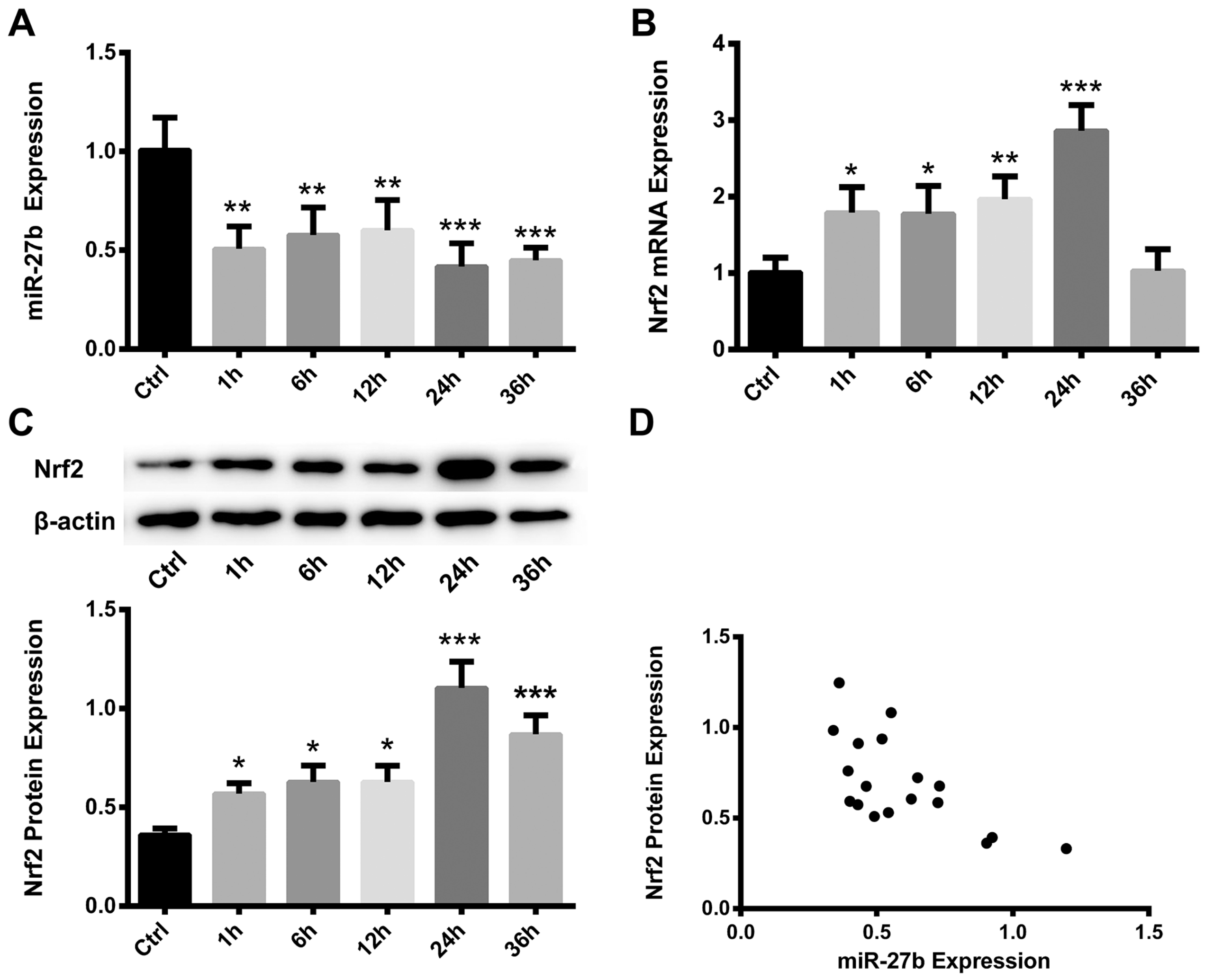

Supplementary Figure 1: Time patterns and negative correlation of miR-27b and Nrf2 expression in FS-treated PC12 cells. Analysis of qRT-PCR data for the expressions of **(A)** miR-27b and **(B)** Nrf2 mRNA. **(C)** Immunoblots and immunoblotting analysis of Nrf2 protein. **(D)** Scatter plot of miR-27b and Nrf2 protein expression ($r=-0.6628$, $p<0.01$). Data are presented as the mean ± SD (n=3). *$p<0.05$, **$p<0.01$, ***$p<0.001$ vs Ctrl.

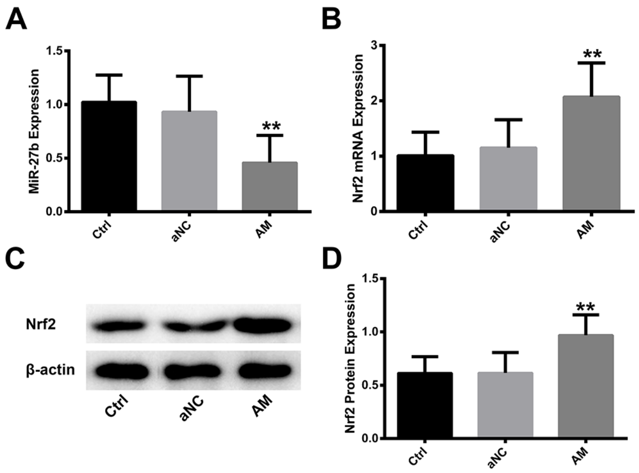

Supplementary Figure 2: ICV injection of miR-27b AM inhibited the endogenous miR-27b and increased Nrf2 expression in rat striatum. Analysis of qRT-PCR data for the expressions of **(A)** miR-27b and **(B)** Nrf2 mRNA. **(C)** Immunoblots and **(D)** immunoblotting analysis of Nrf2 protein. Data are presented as the mean ± SD (n=6). **p<0.01 vs Ctrl.

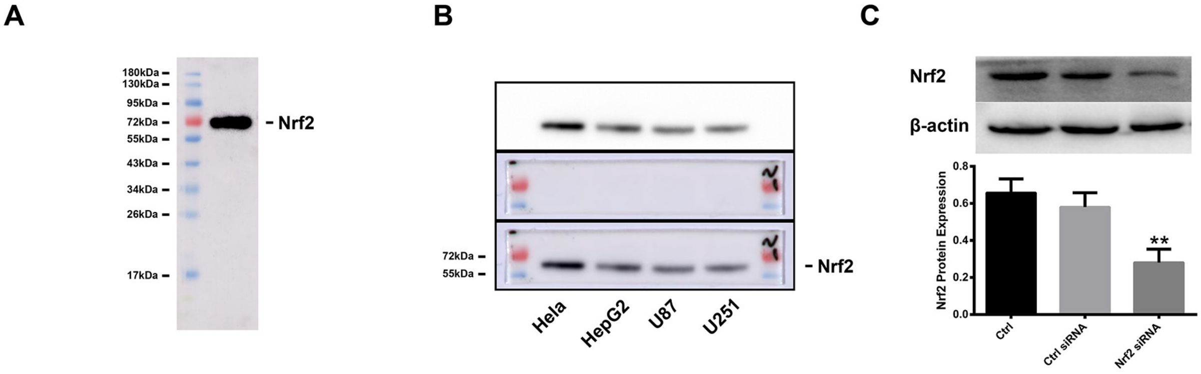

Supplementary Figure 3: The Western blot bands for Nrf2 were specific. (A) Rat brain tissue lysate was subjected to SDS-PAGE, and the blots were visualized using the ECL technique. Only one band at 68 kDa (predicted band size of Nrf2) was detected. **(B)** Whole cell lysates from Hela, HepG2, U87 and U251 cells were used as positive controls. Equal amounts of protein were subjected to SDS-PAGE, and the membrane at 55 - 72 kDa was provided to show the Nrf2 band. PC12 cells were transfected with Nrf2 siRNA or Ctrl siRNA and incubated for 36 h. **(C)** Immunoblots and immunoblotting analysis of the band at 68 kDa. Nrf2 siRNA transfection could significantly reduce the band intensity. The primary antibody was shown to specifically react with Nrf2, and the bands for Nrf2 were specific. Data are presented as the mean ± SD (n=3). **p<0.01 vs Ctrl.

Supplementary Table 1: Sequences of wild-type (WT) and mutant-type (MUT) of Nrf2 3'UTR

**WT**<br>gttcgggaggatggagccttttctgagctagtgtttgttttgtacggctaaaacttcctactgtgatgtgaaatgcagaaacactt<br>tataagtaactatgcagaattatagccaaagctagtgtagaaataatatgaaactttacaaagcattaaagtc<br>tcaatgttgaatcagtttcattttaactctcaagttaatttcttgggcaccatttgggctagtttctgtgtaagtg<br>taaatactacagaacttatttatactgttctcatttgttacagtcatagacttatatgacatctggctaaaagcaaactat<br>tgaaaactaaccaaaccactatactttttttatatactgtatg<br>aacaagaaatgacatttttatattaaattgtttagctctgataaaaattaaaaggagctagcactaataaaggaatatcatgactt
**MUT**<br>gttcgggaggatggagccttttctgagctagtgtttgttttgtacggctaaTTGAAcctTGACACTtgtgaaatgcagaaacact<br>ttataagtaactatgcagaattatagccaaagctagtgtagaaataatatgaaactttacaaagcattaaagtct<br>caatgttgaatcagtttcattttaactctcaagttaatttcttgggcaccatttgggctagtttctgtgtaag<br>tgtaaatactacagaacttatttatactgttctcatttgttacagtcatagacttatatgacatctggctaaaagcaaactat<br>tgaaaactaaccaaaccactatactttttttatatactgtatga<br>acaagaaatgacatttttatattaaattgtttagctctgataaaaattaaaaggagctagcactaataaaggaatatcatgactt

Mutations were generated in the Nrf2 3'UTR sequences complementary to the seed region of miR-27b. The entire 3'UTR (444-bp) of Nrf2 mRNA containing WT or MUT of miR-27b binding sites was amplified and inserted into the pmirGLO vector with XhoI and SacI double digestion. Red words: conserved binding sites of miR-27b. Capital letters: mutation sites.
